# Interpretable Machine Learning-Based Concentric Regional Analysis of OCTA Images for Enhanced Diabetic Retinopathy Detection

**DOI:** 10.3390/bioengineering13040450

**Published:** 2026-04-12

**Authors:** Shrouk Mohamed Osman, Ahmed Alksas, Hossam Magdy Balaha, Ali Mahmoud, Ahmed Gamal, Mohamed El-Said Abdel-Hady, Mohamed Moawad Abdelsalam, Abeer Twakol Khalil, Ashraf Sewelam, Ayman El-Baz

**Affiliations:** 1 Biomedical Engineering Program, Faculty of Engineering, Mansoura University, Mansoura 35516, Egypt; 2 Department of Bioengineering, University of Louisville, Louisville, KY 40292, USA; 3 Mansoura Ophthalmic Center, Mansoura University, Mansoura 35516, Egypt; 4 Department of Computers Engineering and Control Systems, Faculty of Engineering, Mansoura University, Mansoura 35516, Egypt; 5 Department of Electronics and Communications Engineering, Faculty of Engineering, Mansoura University, Mansoura 35516, Egypt; 6 Department of Ophthalmology, Faculty of Medicine, Mansoura University, Mansoura 35516, Egypt

**Keywords:** diabetic retinopathy, ensemble models, explainable AI, Local Interpretable Model-Agnostic Explanations (LIME), optical coherence tomography angiography (OCTA), regional feature extraction, retinal microvasculature

## Abstract

Diabetic retinopathy (DR) remains a major cause of vision loss in patients with diabetes, and earlier recognition of retinal vascular abnormalities may improve risk stratification and clinical follow-up. Optical coherence tomography angiography (OCTA) provides a noninvasive way to visualize the retinal microvasculature and may detect DR-related changes before they are evident on routine clinical assessment. In this work, we investigated whether dividing OCTA images into anatomically defined retinal regions could improve DR classification and clarify which regions carry the greatest discriminative information. The study included 188 OCTA images: 67 from normal eyes, 57 from eyes with mild DR, and 64 from eyes with moderate DR. Each image was divided into seven concentric regions centered on the fovea, and vessel-density features were extracted from each region. Ten machine learning classifiers were trained and compared at the regional level. For each region, the best-performing classifier was retained, and the final prediction was obtained with a majority-voting ensemble. To examine model behavior, Local Interpretable Model-Agnostic Explanations (LIME) were applied. Performance was also compared with that of a transfer-learning MobileNet model trained on whole OCTA images. On the held-out patient-level test set, the ensemble model achieved 97% accuracy, 98% precision, 97% recall, and a 97% F1-score for three-class classification. These results were higher than those obtained with the tested whole-image transfer-learning baselines. The interpretability analysis consistently identified the parafoveal regions as the most informative for classification. Among the seven regions, Region 3 showed the highest overall contribution, followed by Regions 2 and 5, whereas Region 5 became more influential in moderate DR. These results suggest that regional analysis of OCTA-derived vessel density can improve both classification performance and interpretability in DR assessment. The findings also indicate that parafoveal vascular alterations carry substantial discriminative value in distinguishing normal, mild DR, and moderate DR cases. Validation in larger, independent cohorts from multiple centers will be necessary to confirm the generalizability of these findings.

## 1. Introduction

Diabetic retinopathy (DR) is a microvascular complication of diabetes and remains one of the leading causes of visual impairment and blindness worldwide. More than 589 million people are currently living with diabetes, and DR is among its most serious ocular complications. According to the World Health Organization (WHO), DR is responsible for approximately 3.9 million cases of blindness globally. Because early-stage DR often develops without noticeable visual symptoms, timely detection and treatment are critical for preventing avoidable vision loss [1,2].

**Figure 1:**
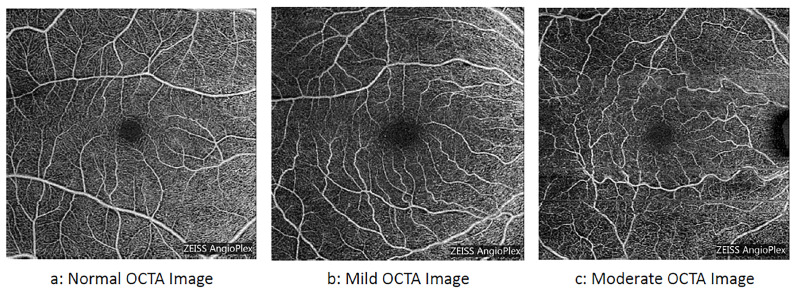
Representative OCTA scans illustrating normal and abnormal retinal vasculature across various DR stages.

Clinically, DR progresses through several stages. Mild non-proliferative diabetic retinopathy (NPDR) is typically characterized by microaneurysms, which indicate early damage to the retinal capillary walls. In moderate NPDR, vascular abnormalities become more pronounced, and areas of capillary nonperfusion begin to appear. As the disease advances to severe NPDR, extensive retinal ischemia develops and stimulates the release of angiogenic factors. In proliferative diabetic retinopathy (PDR), abnormal new blood vessels grow on the retinal surface. These vessels are fragile and susceptible to leakage and hemorrhage, which can result in fibrosis, traction, and severe vision loss [3]. Given this progressive vascular damage, early identification of DR is essential. Sample images for different stages of DR are shown in Figure 1.

Different retinal imaging modalities have been widely used for the detection and evaluation of DR. Each modality offers distinct clinical value for screening, diagnosis, grading, and treatment monitoring across different stages of the disease [3,4].

The four principal imaging modalities used in DR assessment are fundus photography (FP), fluorescein angiography (FA), optical coherence tomography (OCT), and optical coherence tomography angiography (OCTA) [3,4].

**FP** is a non-invasive retinal imaging technique that is widely used to document retinal appearance and detect visible pathological changes. It produces color images based on reflected light from the retina and generates a two-dimensional representation of retinal tissues projected onto an imaging plane [3]. Because of its accessibility and clinical familiarity, FP has long been a standard tool in DR screening. However, it has several limitations, including image distortion, relatively limited resolution for subtle microvascular abnormalities, inability to visualize fine vascular changes across different retinal layers, and a restricted field of view in standard acquisitions [3].

**FA** is an imaging technique used to visualize the retinal vasculature and assess retinal circulation. It is particularly valuable for identifying vascular abnormalities, including microaneurysms, capillary nonperfusion, ischemic areas, neovascularization, and dye leakage that may not be apparent on routine fundoscopy [3,5]. FA is an invasive procedure that requires intravenous injection of sodium fluorescein, typically into an arm vein, followed by sequential image acquisition as the dye circulates through the ocular vasculature [3]. This method provides important information on vessel integrity, permeability, and leakage characteristics. Despite its diagnostic utility, FA has notable drawbacks, including its invasive nature, the time required for image acquisition, and the risk of adverse effects associated with fluorescein injection [3,5].

**OCT** is a non-invasive imaging modality that provides high-resolution cross-sectional images of retinal microstructure. By using low-coherence light backscattering, OCT enables detailed visualization of retinal layers and helps identify structural abnormalities associated with DR [6]. Unlike conventional fundus imaging, OCT offers depth-resolved information and can reveal pathological changes within the retina that are not visible on surface imaging. This technique measures the echo time delay and intensity of reflected light to reconstruct tomographic images of retinal tissue [3,6]. Although OCT is highly effective for structural assessment, it does not provide direct angiographic information and therefore cannot adequately characterize blood flow or vascular leakage. In addition, retinal ischemia, which is an important clinical feature in DR evaluation, cannot be directly assessed using structural OCT alone [3,6].

**OCTA** has emerged as an advanced extension of OCT that addresses several limitations of earlier imaging modalities. OCTA is a relatively recent, non-invasive technique capable of generating high-resolution three-dimensional images of the retinal microvasculature [3]. The method detects motion contrast from flowing blood cells by performing repeated scans at the same retinal location, thereby distinguishing moving particles from static tissue [7]. In contrast to FA, OCTA does not require intravenous dye injection, making it safer and more convenient for repeated clinical use [7,8]. In addition to structural information, OCTA provides quantitative and depth-resolved vascular information, including assessment of blood flow patterns and vessel density across different retinal plexuses [7,8,9,10].

Because of these features, OCTA has become an important imaging modality for DR evaluation. It provides detailed information on both retinal structure and microvasculature, offering advantages over conventional fundus imaging, which does not provide depth-resolved vascular information.

Recent advances in artificial intelligence (AI), particularly machine learning (ML) and deep learning (DL), have improved automated DR detection and classification. These methods support the analysis of retinal images and may assist clinicians in earlier and more consistent diagnosis.

For instance, R. Sathya et al. [11] introduced an agentic-AI-driven framework for the study of diabetic retinopathy. Using retinal fundus images, an AADR-AI can assist in the diagnosis of diabetic retinopathy. 96% accuracy was achieved by using a hybrid CNN-ViT agent and CNN with a ResNet-50 backbone. J Saeidian et al. [12] presented an automated approach to explore the feasibility of FAZ (foveal avascular zone) segmentation and DR classification using optical coherence tomography angiography (OCTA) images.A deep learning pipeline with two steps was created. First, a neural network that combines DeepLabv3+ and EfficientNetB0; second, a convolutional neural network based on Google LeNet that achieves 87% when oversampled for three-class classification (normal, NPDR, and PDR). Le Boité et al. [13] developed an automated approach to identify non-perfusion and low-quality widefield areas in widefield OCTA images to determine the foveal avascular zone (FAZ) area and the retinal non-perfusion index (NPI), two important parameters for diabetic retinopathy (DR) monitoring. obtained good segmentation results (Dice coefficients of 0.879 (FAZ), 0.781 (non-perfusion), and 0.714 (low-quality).

Pradeep et al. [3] applied a ResNet-101-based convolutional neural network (CNN) to OCTA images, achieving high classification accuracy. Li et al. [14] proposed a hybrid hierarchical fusion architecture integrating complementary structural and flow information from OCTA images using dual ResNet branches, attaining an accuracy of 87.86%. El Mostafa et al. [15] developed a multimodal deep fusion framework (SE-ResNet50 and SE-3D-ResNet50) using manifold mixup and squeeze-and-excitation (SE) blocks, achieving 85.66% accuracy. Ebrahimi et al. [16] investigated layer-specific OCTA feature fusion and found that superficial capillary plexus (SCP)-only input achieved the highest accuracy (87.249%). Similarly, Abtahi et al. [17] divided OCTA scans into foveal, parafoveal, and perifoveal regions, extracted region-specific features, and used a Support Vector Machine (SVM) classifier to achieve an accuracy of 87%. Liu et al. [8] used logistic regression with elastic net regularization and reported an accuracy of 82% on OCTA data.

Although these studies support the value of OCTA for DR classification, several limitations remain. Fundus photography and structural OCT do not fully capture the depth-resolved vascular information that may be relevant for detailed DR assessment. In addition, many OCTA-based classification studies emphasize overall performance but provide limited analysis of which retinal regions contribute most strongly to distinguishing early disease stages. This reduces interpretability and may also limit the development of models that are both focused and computationally efficient.

Moreover, because OCTA-based DR datasets are often limited in size and may differ in acquisition protocols, architectures optimized for large-scale whole-image learning may not always be the most data-efficient choice. This consideration motivated our evaluation of anatomically constrained regional descriptors in combination with conventional machine learning classifiers.

**Figure 2:**
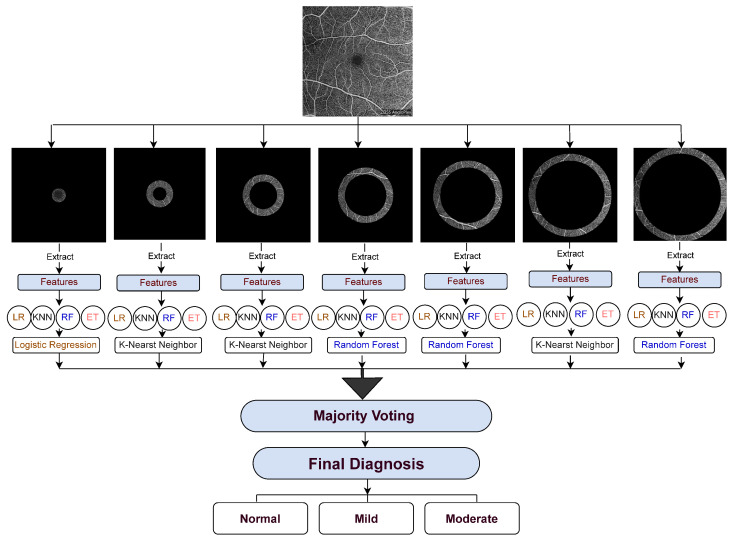
Proposed diagnostic framework for DR detection using segmented OCTA regions and ensemble machine learning classifiers.

To address these gaps, we developed a diagnostic framework based on concentric regional analysis of OCTA images. The aim was to determine which retinal regions and vessel-density features are most informative for three-class DR classification. As shown in Figure 2, the framework integrates regional feature extraction with classifier selection and optimization. The main contributions of this study are as follows:

- We propose a concentric region-based framework for classifying OCTA images into normal, mild DR, and moderate DR.
- We evaluate multiple machine learning classifiers across anatomically defined retinal regions and select the best-performing model for each region to enable region-specific prediction.
- We integrate the selected regional classifiers into a majority-voting ensemble and test the framework on a held-out patient-level test set.
- We use Local Interpretable Model-Agnostic Explanations (LIME) to assess the contribution of different concentric regions and provide an interpretable view of the retinal areas that most influence the final model decisions.

## 2. Materials and Methods

This study used a dataset of 188 OCTA scans to develop a diagnostic model for DR. The dataset included 67 normal cases, 57 mild DR cases, and 64 moderate DR cases. All images were acquired using the ZEISS AngioPlex OCT Angiography system [18], which provides high-resolution, noninvasive imaging of the retinal vasculature. For each scan, the AngioPlex system generated seven image types: one B-scan flow image and six en face vessel density maps.

For the present analysis, the superficial and deep retinal layer maps were selected because of their relevance to DR assessment. Each image had a resolution of 1024×1024 pixels and covered a $6\times 6\;{\mathrm{mm}}^{2}$ area centered on the fovea. The study was designed as a single-center retrospective cross-sectional investigation. All procedures followed the principles of the Declaration of Helsinki. Ethical approval was obtained from the University of Louisville Institutional Review Board (IRB #07.0296). Because the study was retrospective and used data collected during routine clinical care, the requirement for written informed consent was waived by the Institutional Review Board. Data were collected between November 2016 and January 2019, and the analysis was performed from 1 February to 15 April 2019.

We developed a diagnostic pipeline based on the assumption that regional retinal features, particularly those derived from concentric zones around the fovea, contain useful information for early DR detection. The workflow consisted of four stages: (i) preprocessing and concentric region segmentation, (ii) statistical feature extraction using distribution-based metrics, (iii) classification with multiple machine learning models, and (iv) ensemble decision-making with interpretability analysis. An overview of the pipeline is shown in Figure 2.

This study used patient-level partitioning for model development and evaluation to avoid information leakage between training and testing data. The primary evaluation employed an 80:20 patient-level train-test split, and 5-fold cross-validation was performed as an additional robustness analysis.

### 2.1. Data Preprocessing and Concentric Region Division

To extract localized vascular information, each OCTA scan was divided into concentric annular regions centered on the fovea. This approach was based on the assumption that DR-related vascular abnormalities may appear differently across retinal zones. The segmentation procedure was implemented using radii of 25, 50, 75, and 100 pixels to generate concentric regions centered on the fovea.

**Figure 3:**
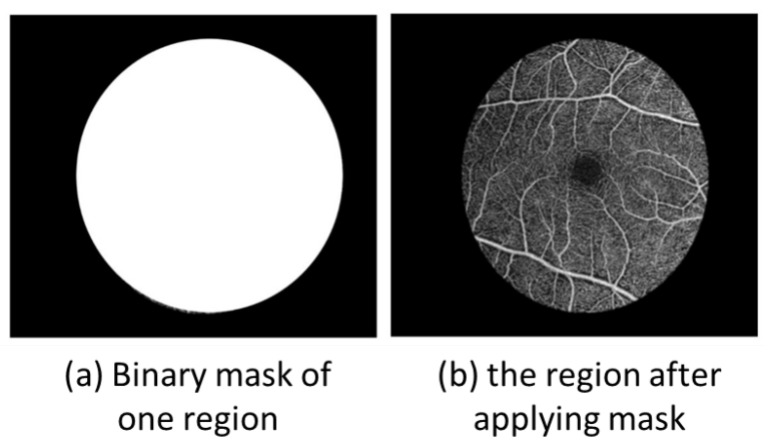
Illustration of binary mask generation and region-wise segmentation of OCTA images.

The total number of regions in each image was determined as:

(1) $$N=\frac{r_{\max}}{R}$$

where *R* denotes the predefined radial step and $r_{\max}$ denotes the maximum Euclidean distance from the foveal center to the image boundary within the OCTA field of view. In practice, $r_{\max}$ defines the outer radial limit used for concentric partitioning of the image. For each annular region, a binary mask was generated to isolate the corresponding pixels for feature extraction [19,20]. This process is shown in Figure 3. The area of the *n*th region was computed as:

(2) $$A_{n}=\pi ({\left(nR\right)}^{2}-{\left(\left(n-1\right)R\right)}^{2})$$

### 2.2. Feature Extraction

After segmentation, local intensity distributions within each region were characterized using the cumulative distribution function (CDF). In this study, CDF-derived percentiles were used to summarize regional differences in pixel intensity, which served as a proxy for local vessel-density variation and contrast differences in the OCTA images [21].

Percentile features for each region were calculated as:

(3) $$P=\frac{n}{N}\times 100\%$$

where *P* represents the percentile, *n* is the rank of a pixel intensity value, and *N* is the total number of pixels within the region. Selected percentiles (e.g., 10th, 20th, 50th, 80th, and 90th) were used to form the feature vector for each image.

### 2.3. Machine Learning Classifiers

The extracted features from each region were used to train ten machine learning classifiers selected to represent diverse model families suitable for structured medical data analysis. These included linear models, instance-based learners, kernel methods, decision trees, bagging ensembles, and boosting ensembles. This broad comparison was intended to determine which classifier type was best suited to the percentile-based regional OCTA features rather than assuming a single model family a priori. The best performing classifier for each region was then assigned to it.

**Logistic regression classifier (LR)**: A supervised machine learning technique. LR is used to obtain an odds ratio when several explanatory variables are present. The LR approach closely resembles multiple linear regression, except that the response variable is binomial. As opposed to linear regression, which predicts continuous values, LR estimates the likelihood that an input is associated with a specific category [22]. Multinomial Logistic Regression: This method is employed when the dependent variable includes three or more categories [23].

**K-Nearest Neighbors (KNN)**: One of the most fundamental basic supervised classification techniques. KNN finds the “k” nearest data neighbors to a specific input and predicts outcomes based on the majority class [24,25].

**Random Forest Classifier (RF)**: A machine learning algorithm that combines tree predictors and many decision trees towards accurate classification. Every tree depends on a random data value. It is an ensemble approach since each tree in the forest is sampled independently with the same distribution. The results are then merged by voting for classification. This helps in increasing accuracy and decreasing errors [26].

**Extra Trees Classifier (ET)**: An ensemble method based on multiple decision trees, in which both the candidate features and split points are randomized during training [27]. In this study, ET was included as a tree-based model with strong variance-reduction capability and good tolerance to noisy feature sets.

**Decision Tree Classifier (DT)**: A supervised non-parametric model that recursively partitions the feature space using decision rules derived from criteria such as entropy or the Gini index [28]. DT was evaluated because it provides a simple baseline and allows straightforward interpretation of the decision structure.

**Adaptive Boosting (AdaBoost)**: An ensemble approach that combines a sequence of weak learners, typically shallow decision trees, by assigning greater weight to samples misclassified in earlier rounds [29]. This iterative reweighting allows the model to focus progressively on more difficult cases.

**Light Gradient Boosting Machine (LGBM)**: A gradient-boosting framework that constructs an additive tree-based model through sequential optimization of the loss function [30]. It was included because of its computational efficiency and its ability to model nonlinear relationships in structured data.

**Extreme Gradient Boosting Classifier (XGB)**: A gradient-boosting method that improves prediction performance by iteratively fitting new trees to the residual errors of the existing model [31]. XGB was evaluated as a high-capacity ensemble model for regional feature-based classification. XGB uses decision trees as base learners and combines them sequentially in a boosting framework. Its objective function includes both prediction loss and regularization terms, which helps improve generalization performance [32].

(4) $${\hat{y}}_{i}=\sum_{j}\theta_{j}x_{ij}$$

where ${\hat{y}}_{i}$ is the predicted output for the *i*th sample, $x_{ij}$ is the value of the *j*th feature, and $\theta_{j}$ is the corresponding feature weight.

**Support Vector Machine (SVM)**: A supervised machine learning-based classification method. It seeks to identify an optimal separating boundary, or hyperplane, between classes in the feature space [33].

**Bagging Classifier (Bg)**: Also referred to as bootstrap aggregation, this ensemble method trains multiple base learners on different resampled subsets of the training data and combines their predictions to reduce variance and improve generalizability [34].

#### Hyperparameter Tuning

Hyperparameters for the machine learning classifiers were selected using Bayesian optimization, followed by a small grid-search refinement around the best-performing configurations. The tuning process considered model-specific parameters such as neighborhood size for KNN, regularization settings for logistic regression and SVM, and tree-ensemble parameters including depth, number of estimators, and learning rate where applicable. The final hyperparameter settings are summarized in Table 1.

### 2.4. Majority Voting and Model Evaluation

**Figure 4:**
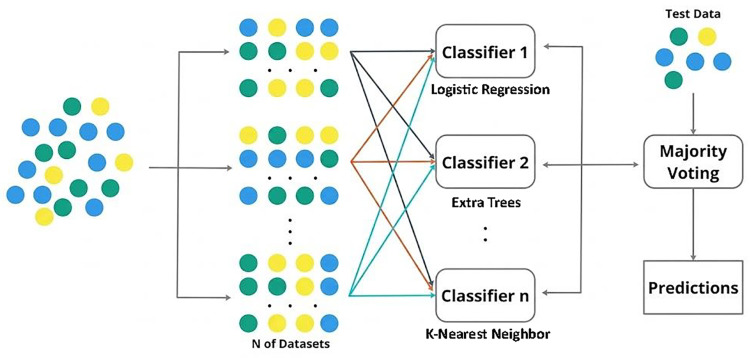
Majority voting ensemble framework aggregating predictions from multiple classifiers.

To improve classification robustness, the predictions of the regions’ selected classifiers were combined using a majority-voting ensemble [35]. The final class label $\hat{y}$ was determined as:

(5) $$\hat{y}=\mathrm{mode}\left\{C_{1}\left(x\right),C_{2}\left(x\right),\ldots ,C_{m}\left(x\right)\right\}$$

where *x* denotes the feature vector extracted from the OCTA image for the region being evaluated, $C_{j}\left(x\right)$ is the class prediction of the *j*-th selected classifier for that input, and *m* is the total number of regional classifiers included in the voting scheme. Figure 4 illustrates the utilized majority voting ensemble framework.

Model performance was evaluated using standard classification metrics, including F1-score, recall, precision, and accuracy. These metrics are defined as:

(6) $$F1\text{-}\mathrm{score}=2\times \frac{\mathrm{Precision}\times \mathrm{Recall}}{\mathrm{Precision}+\mathrm{Recall}}$$

(7) $$\mathrm{Recall}=\frac{\mathrm{TP}}{\mathrm{TP}+\mathrm{FN}}$$

(8) $$\mathrm{Precision}=\frac{\mathrm{TP}}{\mathrm{TP}+\mathrm{FP}}$$

(9) $$\mathrm{Accuracy}=\frac{\mathrm{TP}+\mathrm{TN}}{\mathrm{TP}+\mathrm{TN}+\mathrm{FP}+\mathrm{FN}}$$

A confusion matrix was also constructed for each classifier to visualize prediction performance across class labels [36,37].

### 2.5. Train-Test Split vs. K-Fold Cross-Validation

**Figure 5:**
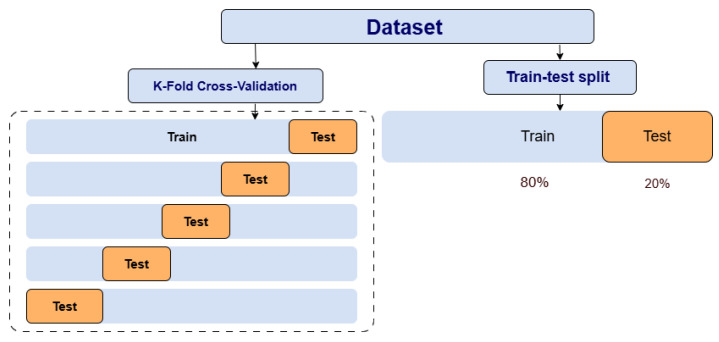
The structural differences between train-test split and K-fold cross-validation.

The train-test split approach splits the dataset into simply two sections: one for training and one for testing, whereas K-fold cross-validation splits the dataset into several subsets to train and test the model iteratively. Although the train-test split method is simple to apply and fast to compute, its performance estimates can vary considerably depending on how the dataset is divided. K-fold cross-validation gives a more robust and reliable performance assessment by limiting the effects of data variability. Utilizing several training and testing cycles reduces the risk of molding to a certain data split [38]. Figure 5 illustrates the structural differences between these two methods. Here we will take k as 5.

### 2.6. Model Interpretability Using LIME

To improve post hoc interpretability of the ensemble classifier, we used the Local Interpretable Model-Agnostic Explanations (LIME) framework [39]. LIME fits sparse linear models to approximate the local decision behavior of black-box classifiers and provides feature-level explanations for individual predictions.

LIME was applied to vessel density features extracted from seven concentric annular regions spanning a radius of 0 to 525 pixels, with 75-pixel intervals between regions. No dimensionality reduction was performed, so the original feature space was retained.

Global feature importance was quantified by averaging the absolute LIME weights across all subjects. Region 3 (150–225 px) had the largest overall contribution (21.04%), followed by Region 2 (16.53%) and Region 5 (15.52%). In contrast, the peripheral regions (Regions 6–7) and the central region (Region 1) showed lower average importance, with values of approximately 11–12%.

The class-specific analysis showed a similar pattern. Region 3 remained the most influential region in all three classes and reached its highest contribution in Moderate DR (22.14%). Region 5 also became more prominent in Moderate DR, where its average importance increased to 18.26%. These findings indicate that the intermediate retinal regions carried the strongest discriminative signal in this dataset.

## 3. Results

To preserve clinical relevance and minimize data leakage, all experiments were performed with patient-level partitioning; images from the same patient were not split across the training, validation, and test sets. The main evaluation metrics for the ensemble model were then computed on a held-out test set. In addition, a 5-fold cross-validation analysis was performed to assess the stability of the results across different splits.

### 3.1. Region Optimization and Classifier Evaluation

**Figure 6:**
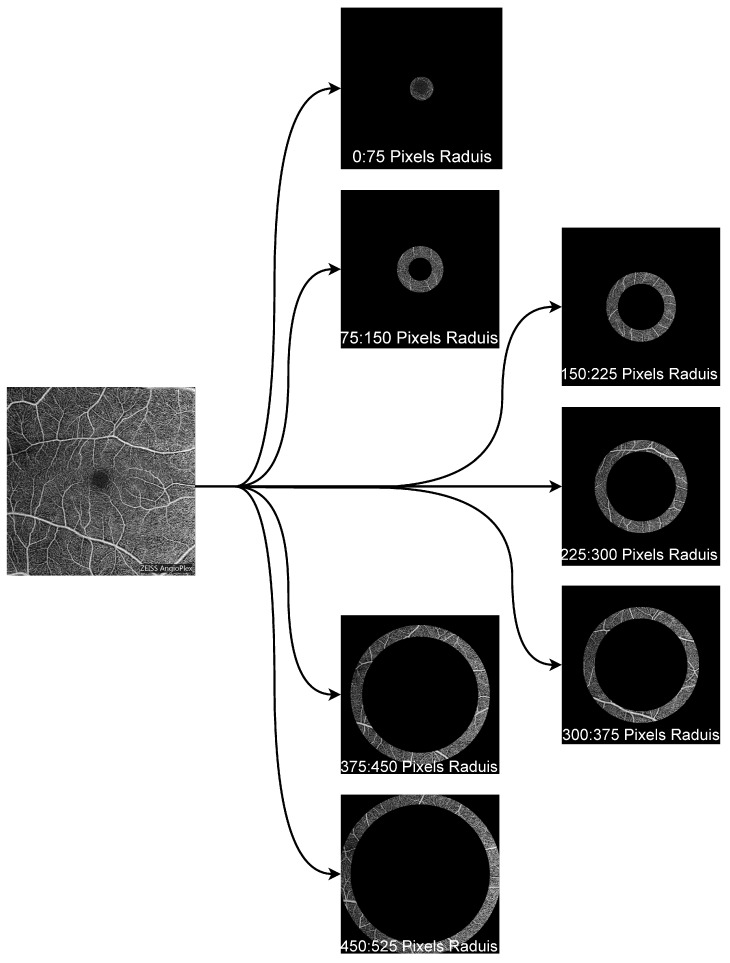
Illustration of the seven concentric regions within an OCTA image, centered on the fovea.

To determine an appropriate regional configuration for OCTA feature extraction, we compared several candidate radii empirically. Partitioning each 1024×1024 image into seven concentric annular regions with a fixed radial increment of R=75 pixels gave the best trade-off between spatial resolution and classification performance. This segmentation scheme is visualized in Figure 6.

**Figure 7:**
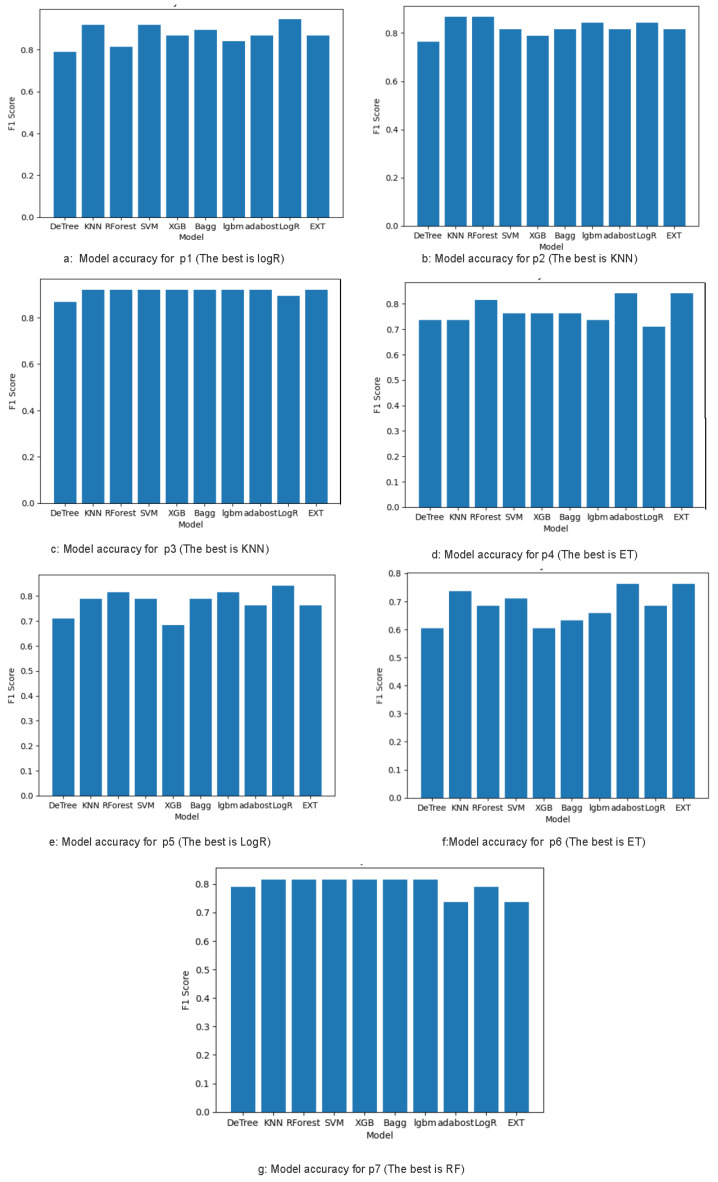
Model performance comparison for each region.

From each region, nine percentile-based features were extracted, producing seven separate datasets. Each was evaluated using ten classifiers: KNN, LR, ET, SVM, DT, XGB, RF, LGBM, AdB, and Bg. The classifiers’ performance comparison is shown in Figure 7 However, we concentrate only on the four top-performing classifiers: logistic regression, K-Nearest Neighbor, Extra Trees, and Random Forest, as shown in Table 2.

Table 2 presents the classification results per region. **The LR** classifier achieved the best performance in R1 and R5 (the innermost and outermost zones), with accuracies of 95% and 84%, respectively. **The KNN** classifier performed best in R2 and R3 with accuracies of 87% and 93%, respectively. **The ET** classifier was optimal in the mid-peripheral zones of R4 and R6 with accuracies of 82% and 76%, respectively. **The RF** classifier achieved the best performance in R7 with an accuracy of 82%. These results suggest that different regions highlight distinct vascular cues, warranting a region-specific classification strategy.

### 3.2. Majority Voting and Overall Model Performance

To consolidate the region-specific strengths of individual classifiers, a majority voting ensemble strategy was employed. For each OCTA image, predictions from the best-performing classifier per region were aggregated, and the final class label was assigned based on the most frequent predicted label across the seven regions.

**Figure 8:**
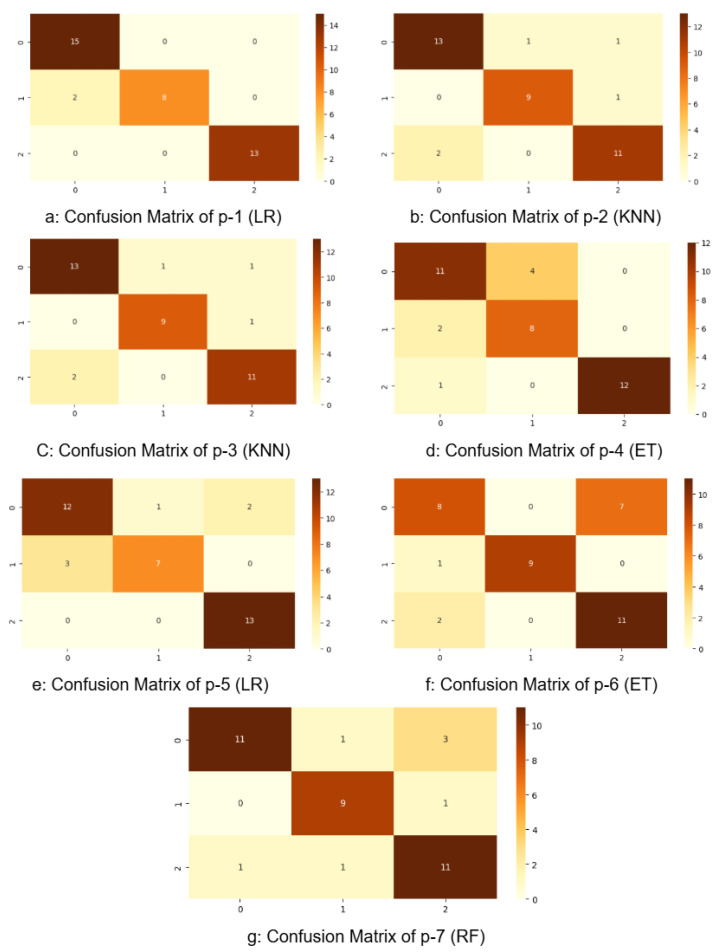
Confusion matrix illustrating classification performance across individual regions. Diagonal values show the correct predictions for each DR class.

**Figure 9:**
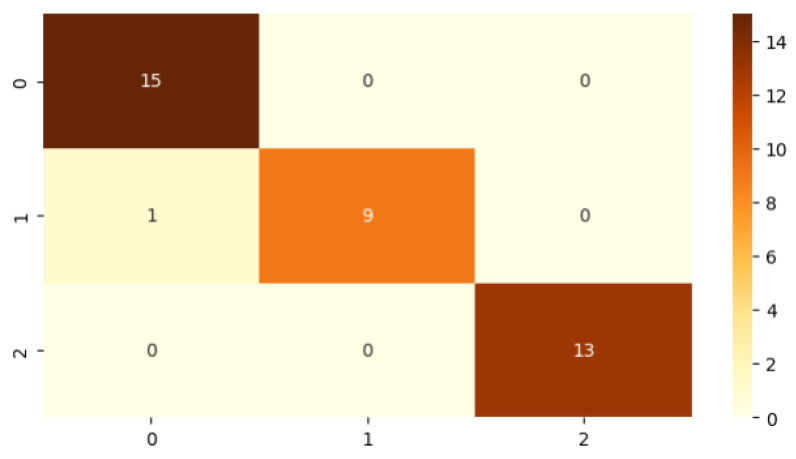
Confusion matrix for the proposed ensemble model using majority voting. Strong diagonal values indicate robust classification across DR stages.

Table 3 summarizes the best classifiers for each region and their individual metrics. Notably, the ensemble model achieved substantial improvements over region-specific models, with an F1-score of **97%**, recall of **97%**, precision of **98%**, and an overall accuracy of **97%**. The corresponding confusion matrices for the individual-region classifiers and the proposed ensemble model are shown in Figure 8 and Figure 9, respectively. These results show the ability of our multi-classifier fusion approach in capturing complementary diagnostic cues across anatomical zones.

### 3.3. Comparison of Individual vs. Ensemble Performance

**Figure 10:**
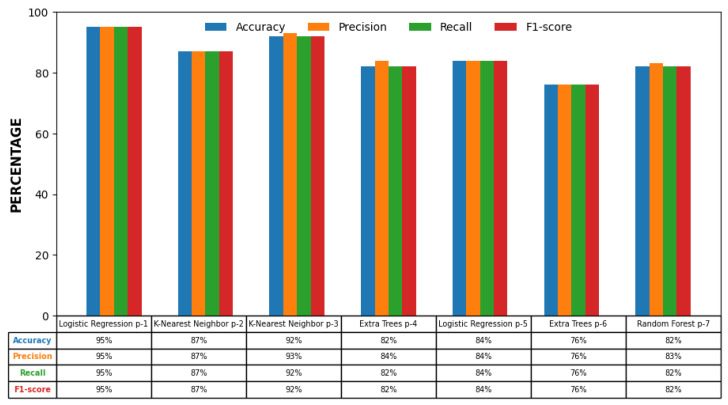
Comparison of performance metrics for individual regions versus the majority voting ensemble. The ensemble consistently outperforms all single-region classifiers.

Figure 10 visualizes the comparative performance of individual region-based classifiers against the proposed ensemble approach. The majority voting strategy consistently outperforms the best-performing single-region models across all evaluation metrics, demonstrating the value of spatial integration and classifier diversity in improving diagnostic accuracy.

**Figure 11:**
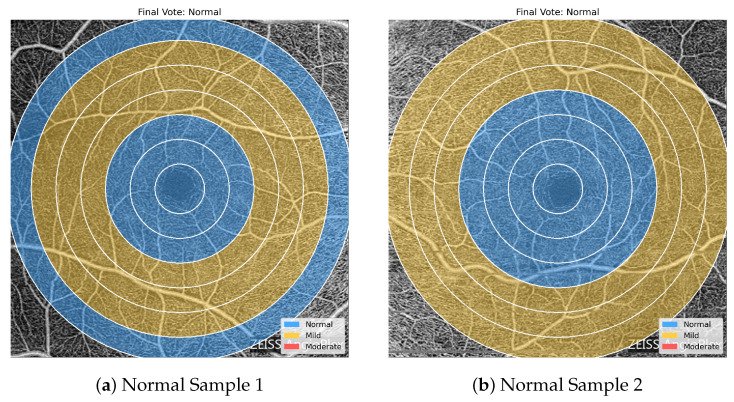
Sample overlays for two OCTA images assigned to the Normal class after majority voting. Although some surrounding regions are predicted as Mild, the dominant regional pattern remains consistent with a Normal image-level classification, illustrating the stabilizing effect of the voting scheme.

**Figure 12:**
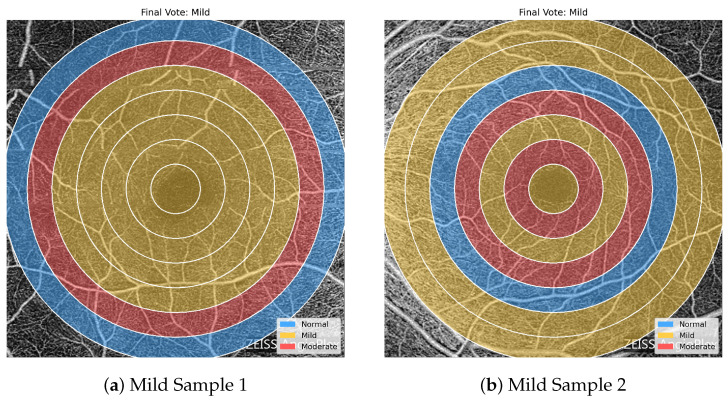
Representative overlays for two OCTA images assigned to the Mild class after majority voting. The regional predictions are heterogeneous and include Normal, Mild, and Moderate labels, showing how the final decision is determined by the dominant regional trend rather than by complete regional agreement.

**Figure 13:**
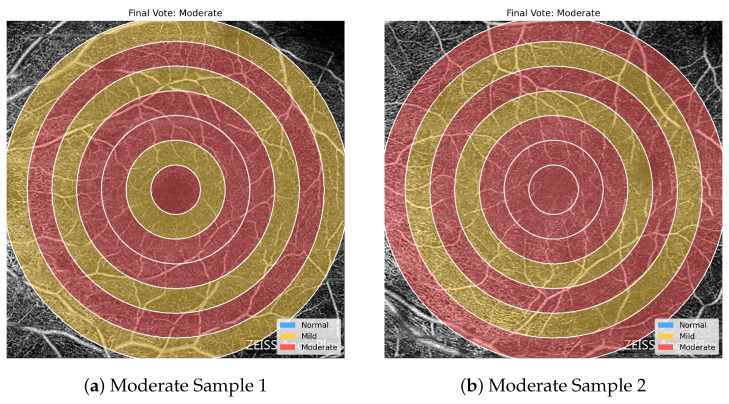
Representative overlays for two OCTA images assigned to the Moderate class after majority voting. In both cases, Moderate-labeled regions are sufficiently prevalent to determine the final image-level prediction despite the presence of some less severe regional assignments.

Figure 11, Figure 12 and Figure 13 highlight the value of the majority-voting scheme used in the proposed framework. The regional predictions within a single OCTA image are not always uniform, and neighboring severity levels may appear simultaneously across different concentric zones. Nevertheless, the final image-level label is determined by the dominant regional pattern rather than by any single ring. This is clearly seen in the Normal and Mild examples, where mixed regional assignments are still resolved into the appropriate final class, while the Moderate examples show a stronger accumulation of Moderate-labeled regions that drives the final decision toward the more severe category. These examples demonstrate how voting stabilizes the classification outcome and makes the prediction less sensitive to local regional variability.

### 3.4. Comparison Between the K-Fold Cross-Validation Approach and the Train-Test Split Approach

To reliably estimate the proposed machine learning model’s accuracy, we compare the test split approach results and the k-fold cross-validation approach results. Choosing K = 5 is a popular choice because it offers an optimal balance between computational efficiency and the assessment of model performance. Table 4 evaluates the performance of each fold over all regions and compares the results with our proposed model. The cross-validation analysis supports the stability of the proposed framework across different data partitions and complements the held-out test set results.

### 3.5. Comparison with Deep Learning Baseline

For deep-learning benchmarking, we evaluated whole-image transfer-learning baselines using the same patient-level 80:20 split as the machine learning experiments. The MobileNet model was trained using categorical cross-entropy loss and the Adam optimizer, with data augmentation applied only to the training subset. In supplementary transfer-learning experiments performed on the same split, ResNet and EfficientNet baselines were also evaluated; however, these additional CNN models likewise yielded lower performance than the proposed regional ensemble. This comparison evaluates the efficiency, interpretability, and diagnostic accuracy of the ensemble model relative to a conventional convolutional neural network (CNN) pipeline.

**Data Augmentation:** Due to the relatively small and imbalanced dataset, image augmentation techniques were applied to increase sample variability. Transformations included random rotation, zooming, flipping, and contrast adjustment, aiming to prevent overfitting and simulate natural acquisition variability.

**Figure 14:**
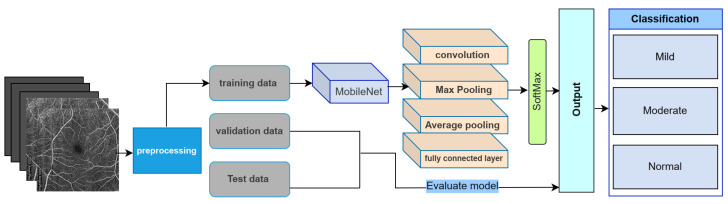
Deep learning pipeline with CNN layers applied to OCTA images. Includes data augmentation, feature extraction, and classification stages.

**Figure 15:**
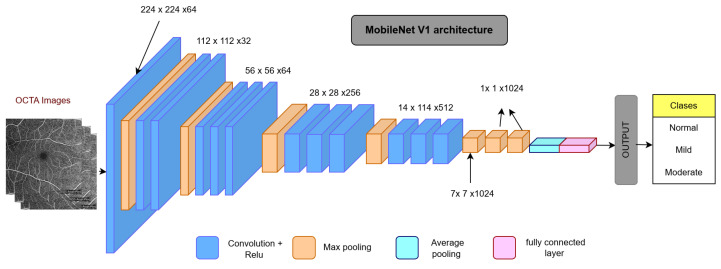
MobileNet structure used in the transfer learning model. Designed for low computational cost and high efficiency in image classification.

**Model Architecture:** The CNN architecture (Figure 14) consisted of standard layers—input, convolution, pooling, fully connected (FC), and output—optimized for retinal image processing. MobileNet, selected for its compact architecture and low parameter count, was used as the backbone model (Figure 15).

**Transfer Learning:** To avoid training from scratch and leverage high-quality pretrained features, ImageNet-trained weights were transferred into the MobileNet layers. Fine-tuning was performed using the OCTA image dataset, with early layers frozen and deeper layers adapted to DR-specific patterns.

**Figure 16:**
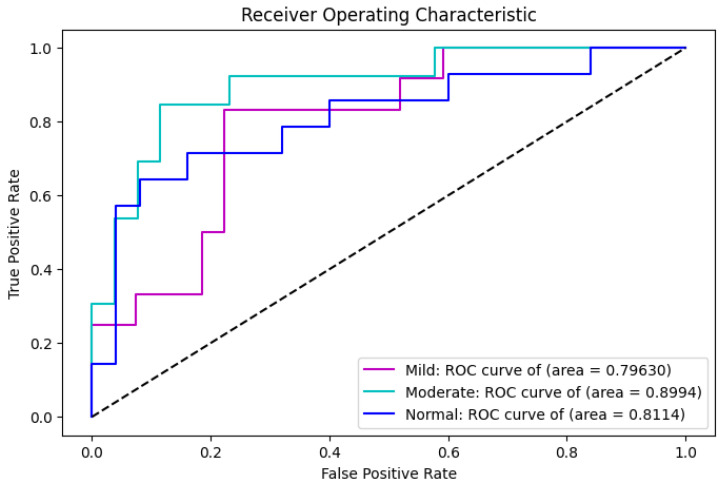
ROC curve for the deep learning MobileNet classifier, showing area under the curve (AUC) performance across DR stages.

**Model Performance:** The MobileNet-based CNN achieved an overall classification accuracy of 71%, with F1-score, recall, and precision values of 72%, 72%, and 73%, respectively. The specificity of the CNN model is 86% and the sensitivity is 71%. These results are visualized in the receiver operating characteristic (ROC) curve in Figure 16, which shows moderate separation between DR stages.

**Performance Comparison:** Table 5 presents the quantitative comparison between the deep learning baseline and our proposed ensemble model. The proposed ensemble outperformed the tested MobileNet baseline while also offering greater interpretability through anatomically localized feature analysis. The superior performance of the ensemble relative to the tested MobileNet baseline may reflect the limited dataset size and the advantage of anatomically guided feature extraction in this setting.

### 3.6. Model Interpretability via Regional LIME Analysis

To examine how the proposed ensemble model used regional information for DR classification, we applied Local Interpretable Model-Agnostic Explanations (LIME). This analysis was used to characterize region-level contributions at both the individual-subject and overall dataset levels.

**Regional Setup:** Each OCTA image was divided into seven concentric annular regions (R1–R7), using a fixed radial interval of 75 pixels from the foveal center (0–525 px). LIME was then applied to the vessel density features extracted from these regions to generate local explanations for the ensemble model predictions.

**Figure 17:**
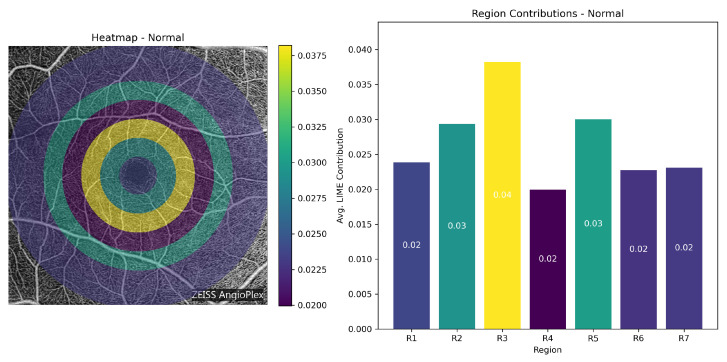
LIME visualization for a Normal case. **Left**: region-wise contribution heatmap overlaid on the OCTA image. **Right**: bar plot of regional contributions.

**Figure 18:**
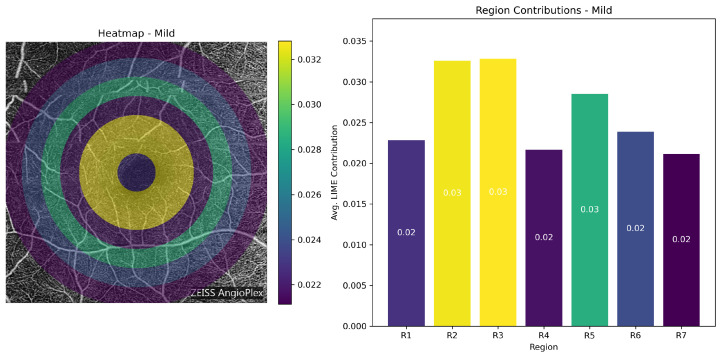
LIME visualization for a Mild DR case. Region 2 shows a relatively larger contribution, consistent with early parafoveal microvascular change.

**Figure 19:**
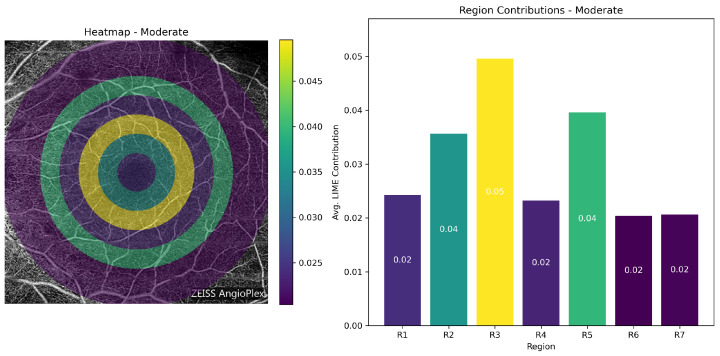
LIME visualization for a Moderate DR case. Region 5 shows increased contribution, indicating greater influence of more peripheral vascular abnormalities.

**Figure 20:**
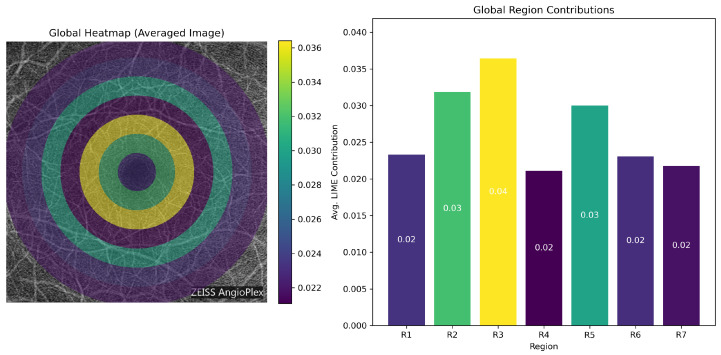
Average LIME contribution across all subjects. Region 3 (150–225 px) shows the highest overall contribution.

**Class-wise Heatmap Analysis:** Figure 17, Figure 18, Figure 19 and Figure 20 show representative examples from each class together with the corresponding LIME heatmaps and bar plots. Across these examples, several class-related patterns were observed:

- In **Normal** eyes, the model placed greater emphasis on Regions 2 and 3 (75–225 px), corresponding to the parafoveal area.
- In **Mild DR**, Region 2 became more prominent, suggesting that early class separation was influenced by parafoveal microvascular change.
- In **Moderate DR**, the contribution of Region 5 (300–375 px) increased, indicating a greater role for outer retinal regions in the final classification.

**Quantitative Attribution Summary:** Table 6 summarizes the average LIME weights for each region across all classes. Region 3 showed the highest overall contribution to classification (21.04%), followed by Region 2 and Region 5. In contrast, the foveal center (R1) and the outer regions (R6–R7) contributed less.

**Figure 21:**
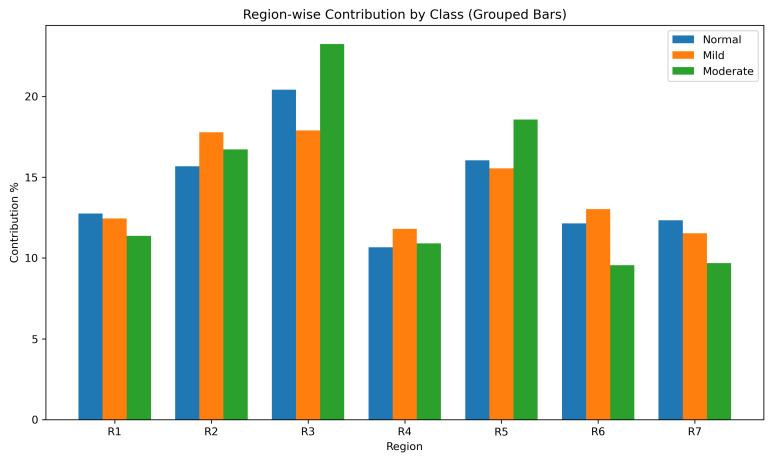
Grouped bar chart of regional LIME contributions for the Normal, Mild, and Moderate classes. Regions 2, 3, and 5 show the largest contributions overall.

**Figure 22:**
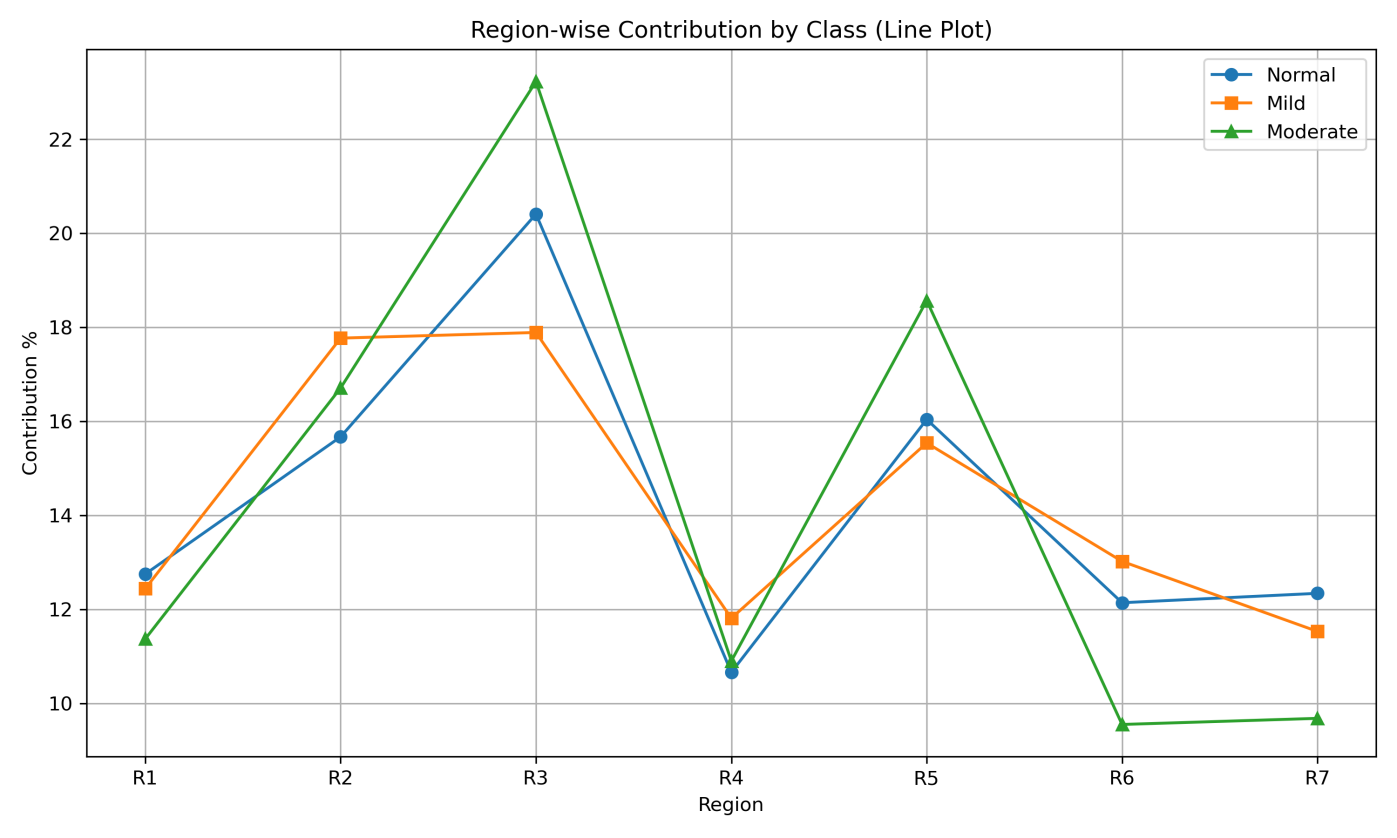
Line plot of LIME contribution patterns across retinal regions for each class. The plot shows shifts in regional importance with increasing DR severity.

**Inter-Class Visualizations:** Figure 21 presents a grouped bar chart of regional contributions for each class. Region 3 remained highly influential across all classes, whereas Region 5 showed greater importance in Moderate DR. The line plot in Figure 22 shows the same trend and illustrates how the relative contribution of retinal regions changed with disease severity.

**Interpretability Insights:** The LIME analysis showed that the ensemble did not rely uniformly on all retinal regions. Instead, the model assigned greater weight to specific concentric zones, and the relative contribution of these zones varied across the three classes. This pattern indicates that the classification decisions were driven by localized spatial information rather than by a uniform summary of the full OCTA image.

## 4. Discussion

In this study, we evaluated a region-based machine learning framework for early DR detection and classification using OCTA scans. By dividing each image into concentric zones centered on the fovea, the analysis aimed to capture spatial differences in retinal vascular alteration across disease stages. The results showed that this regional approach improved classification performance, particularly when region-specific models were combined in an ensemble and examined with post hoc interpretability analysis.

The main performance estimates were obtained from a held-out patient-level test set, with 5-fold cross-validation included as an additional robustness analysis. Although patient-level partitioning reduced the risk of data leakage, the dataset remained relatively small and was derived from a single cohort, which may restrict the generalizability of the findings.

### 4.1. Region-Specific Diagnostic Insights

The region-wise analysis showed that the retinal zones did not contribute equally to classification. Region 3 (150–225 pixels), corresponding to the parafoveal area, provided the strongest and most consistent contribution across classes. Region 1 (0–75 pixels), which includes the foveal avascular zone (FAZ), also contributed to classification, although its average importance was lower than that of Region 3 in the LIME analysis. This distribution is consistent with the known spatial pattern of early DR-related microvascular change, which often involves capillary non-perfusion, microaneurysm formation, and disruption of the parafoveal capillary network. Alterations in FAZ morphology may also contribute, but in our dataset they appeared to be less discriminative than the intermediate parafoveal regions.

In the Moderate DR class, Region 5 (300–375 pixels) became more influential, suggesting that vascular abnormalities extended beyond the central macular region as disease severity increased. By contrast, Region 6 (375–450 pixels) contributed less across classes, indicating a weaker role in the final classification decisions within this dataset.

### 4.2. Model Interpretability and Clinical Relevance

LIME provided a region-level view of how the ensemble classifier reached its predictions. The heatmaps and attribution scores suggested that the model relied on spatial patterns that were broadly consistent with known retinal vascular changes in DR. In particular, the analysis indicated a shift from stronger parafoveal emphasis in earlier disease toward greater contribution from more peripheral regions in Moderate DR.

These findings may also be useful for future biomarker development. The relatively strong contribution of Regions 2 and 3 suggests that these areas may be useful targets for feature design in later studies. In addition, the regional attribution profiles may help guide more focused imaging or analysis strategies, although this would need to be evaluated prospectively.

### 4.3. Algorithmic Design Considerations

The regional classifier results and the LIME findings should be interpreted as related but not interchangeable. Some concentric regions showed stronger standalone classification performance, whereas LIME identified Region 3 as the largest overall contributor to the final ensemble decision, followed by Regions 2 and 5. Region 5 showed greater importance in Moderate DR. This difference is consistent with the distinction between the two analyses: standalone regional classification evaluates each region independently, whereas LIME estimates how much that region contributes within the full ensemble model.

The comparative analysis further showed that classifier performance was not uniform across retinal regions. Logistic regression performed better in the central regions, K-nearest neighbor in the more peripheral regions, extra trees in the intermediate regions, and random forest near the image border. This pattern supports the use of a heterogeneous ensemble, since different classifiers appeared to capture complementary characteristics of the regional feature sets. The final ensemble achieved an accuracy of 97%, outperformed the tested MobileNet baseline, and remained easier to interpret while requiring fewer samples. This performance advantage of the proposed framework over the tested whole-image transfer-learning CNN baselines may reflect several factors. First, the concentric regional decomposition imposes an anatomically meaningful structure on the OCTA data and allows the model to focus on localized vascular differences that may be diluted in whole-image analysis. Second, the use of compact percentile-based regional descriptors reduces feature dimensionality, which may be advantageous in a relatively small three-class dataset. Third, the majority-voting strategy integrates complementary information across retinal regions and stabilizes the final prediction when individual regional predictions differ.

### 4.4. Limitations

This study has several limitations. First, the dataset was relatively small and originated from a single center, which may limit the generalizability of the reported performance. In addition, an external validation cohort was not available for the present revision. Although patient-level partitioning and cross-validation were used to reduce overfitting and information leakage, the reported performance should still be interpreted as a single-cohort result. Future studies should evaluate the framework in larger independent multi-center cohorts and across a broader range of DR severity levels.

Second, the analysis was limited to static OCTA scans. Because DR is a progressive disease, longitudinal data could provide additional insight into temporal vascular changes. The present study also relied only on vessel density features. Including other OCTA-derived biomarkers, such as FAZ morphology or perfusion-related measures, may improve predictive performance.

Third, although LIME was used to improve interpretability, it is based on local linear approximations and may not fully represent nonlinear relationships learned by ensemble models. Other interpretability methods, such as SHAP, could provide complementary information in future work.

Finally, the use of traditional machine learning models improved efficiency and interpretability, but it also limited the ability to learn features automatically from the images themselves. These limitations suggest several directions for future work, including larger multicenter datasets, multimodal imaging, and longitudinal modeling.

### 4.5. Broader Impact

The ability to identify retinal regions that contribute most strongly to classification may improve the clinical interpretability of AI-based OCTA analysis. By relating model output to anatomically defined retinal zones, the proposed framework offers a more transparent basis for prediction than a whole-image classification result alone. In practice, such an approach could support screening and clinical review by indicating which areas of the OCTA scan were most influential in the final decision.

## 5. Conclusions and Future Work

This study presented a region-based machine learning framework for early DR detection using OCTA images. Each image was divided into seven concentric regions centered on the fovea, allowing vessel-density features to be extracted in a spatially organized way and enabling analysis of localized microvascular variation associated with DR.

The results showed that retinal regions differed in predictive value and that classifier performance was not uniform across regions. On the basis of these findings, we combined the best-performing regional classifiers in a majority-voting ensemble, which achieved 97% F1-score, 97% recall, 98% precision, and a 97% accuracy. Relative to the tested MobileNet baseline, the proposed framework showed stronger overall performance while also providing clearer regional interpretability.

An additional contribution of this study was the use of LIME to examine how regional information influenced classification. The LIME analysis showed that the model relied most strongly on the parafoveal region (Region 3), with Region 5 becoming more influential in Moderate DR. These findings indicate that the ensemble did not treat all retinal zones equally and that the most informative regions were anatomically localized rather than uniformly distributed across the image.

### Future Work

Future work can extend this framework in several directions:

- **Multi-modal data integration:** Combining OCTA with other retinal imaging modalities, such as color fundus photography or fluorescein angiography, may improve characterization of vascular and structural abnormalities across DR stages.
- **Deep learning with regional guidance:** Although the current ensemble performed well on the available dataset, future models could incorporate anatomically guided attention or region-aware feature learning within an end-to-end deep-learning framework.
- **Longitudinal analysis:** Applying the method to longitudinal OCTA data may help identify imaging markers associated with disease progression and temporal change.
- **Prospective and multicenter validation:** Additional evaluation in larger, independent, and prospectively collected cohorts will be necessary to determine the robustness and generalizability of the framework in clinical settings.
- **Additional interpretability methods:** Future studies may compare LIME with other interpretability approaches, such as SHAP, to examine whether the regional importance patterns remain consistent across methods.

In summary, the findings support the use of region-aware and interpretable machine learning for OCTA-based DR classification. The proposed framework combined strong classification performance with a transparent regional analysis of model behavior. Further validation will be needed, but the results suggest that anatomically localized OCTA analysis may be a useful direction for clinically interpretable DR assessment.

## Figures and Tables

**Table 1:** Optimal hyperparameter values for the proposed models.

Algorithm	Hyperparameter	Optimal Value
KNN	Number of neighbors (*K*)	3, 7
	Distance metric	2
LR	*C*	1
	Penalty	L2
	Maximum iterations	100
SVM	*C*	1
	γ	scale
	Kernel	RBF
RF	Number of estimators	100
	Random state	42
DT	Maximum depth	80
	Criterion	entropy
ET	Number of estimators	100
	Maximum depth	10
XGBoost	Number of estimators	80
	Maximum depth	8
	Learning rate	0.05
AdaBoost	Base estimator	Decision Tree
	Number of estimators	100
LGBM	Number of leaves	31
	Number of estimators	100
	Maximum depth	1
Bagging	Base estimator	Decision Tree
	Number of estimators	50
	Bootstrap	True

**Table 2:** Evaluation of classifiers for individual concentric regions.

Region	Diameter (Pixels)		Classifier	Evaluation Metrics			
	Inner	Outer		Accuracy	Precision	Recall	F1-Score
R1	0	75	**Logistic Regression**	**95%**	**95%**	**95%**	**95%**
			K-Nearest Neighbor	92%	93%	92%	92%
			Random Forest	79%	79%	79%	79%
			Extra Trees	87%	86%	87%	87%
R2	75	150	Logistic Regression	84%	84%	84%	84%
			**K-Nearest Neighbor**	**87%**	**87%**	**87%**	**87%**
			Random Forest	87%	86%	87%	87%
			Extra Trees	82%	82%	82%	82%
R3	150	225	Logistic Regression	89%	90%	89%	89%
			**K-Nearest Neighbor**	**92%**	**93%**	**92%**	**92%**
			Random Forest	92%	93%	92%	92%
			Extra Trees	92%	93%	92%	92%
R4	225	300	Logistic Regression	71%	72%	71%	71%
			K-Nearest Neighbor	74%	76%	74%	74%
			Random Forest	82%	83%	82%	82%
			**Extra Trees**	**82%**	**84%**	**82%**	**82%**
R5	300	375	**Logistic Regression**	**84%**	**84%**	**84%**	**84%**
			K-Nearest Neighbor	79%	78%	79%	79%
			Random Forest	82%	82%	82%	81%
			Extra Trees	76%	76%	76%	76%
R6	375	450	Logistic Regression	68%	71%	68%	69%
			K-Nearest Neighbor	74%	76%	74%	74%
			Random Forest	63%	66%	63%	64%
			**Extra Trees**	**76%**	**76%**	**76%**	**76%**
R7	450	512	Logistic Regression	79%	83%	79%	79%
			K-Nearest Neighbor	82%	82%	82%	82%
			**Random Forest**	**82%**	**83%**	**82%**	**82%**
			Extra Trees	79%	80%	79%	79%

Note: Bold indicates the selected best-performing classifier for each region.

**Table 3:** Evaluation of the proposed Majority Voting model versus individual region-wise classifiers.

Region	Diameter (Pixels)		Best Classifier	Evaluation Metrics			
	Inner	Outer		Accuracy	Precision	Recall	F1-Score
R1	0	75	Logistic Regression	95%	95%	95%	95%
R2	75	150	K-Nearest Neighbor	87%	87%	87%	87%
R3	150	225	K-Nearest Neighbor	92%	93%	92%	92%
R4	225	300	Extra Trees	82%	84%	82%	82%
R5	300	375	Logistic Regression	84%	84%	84%	84%
R6	375	450	Extra Trees	76%	76%	76%	76%
R7	450	512	Random Forest	82%	83%	82%	82%
**Proposed Model (Majority Voting)**				**97%**	**98%**	**97%**	**97%**

Note: Bold indicates the best-performing model.

**Table 4:** A comparison between the k-fold approach and our proposed model relative to accuracy.

Region	k Fold = 5	Accuracy	Average Accuracy	Proposed Model Accuracy
R1	Fold 1	94%	89%	95%
	Fold 2	89%		
	Fold 3	71%		
	Fold 4	94%		
	Fold 5	97%		
R2	Fold 1	84%	89%	87%
	Fold 2	92%		
	Fold 3	94%		
	Fold 4	86%		
	Fold 5	91%		
R3	Fold 1	92%	88%	92%
	Fold 2	89%		
	Fold 3	81%		
	Fold 4	91%		
	Fold 5	89%		
R4	Fold 1	73%	80%	82%
	Fold 2	79%		
	Fold 3	84%		
	Fold 4	72%		
	Fold 5	91%		
R5	Fold 1	81%	87%	84%
	Fold 2	86%		
	Fold 3	78%		
	Fold 4	91%		
	Fold 5	97%		
R6	Fold 1	73%	74%	76%
	Fold 2	76%		
	Fold 3	71%		
	Fold 4	78%		
	Fold 5	72%		
R7	Fold 1	81%	85%	82%
	Fold 2	86%		
	Fold 3	81%		
	Fold 4	91%		
	Fold 5	83%		

**Table 5:** Performance comparison: Deep learning vs. proposed machine learning ensemble.

Method	Accuracy	Precision	Recall	F1-Score
Deep Learning (MobileNet)	71%	73%	72%	72%
Proposed ML Ensemble	**97%**	**98%**	**97%**	**97%**

Note: Bold indicates the selected best-performing method.

**Table 6:** Average region-wise LIME contributions across all subjects. Values are reported as percentage contributions to the model prediction.

Region	Global Avg.	Global %	Normal %	Mild %	Moderate %
R1	0.0238	12.33	12.42	12.79	9.83
R2	0.0319	16.53	14.75	17.37	18.13
R3	0.0406	21.04	22.72	19.49	22.14
R4	0.0217	11.24	11.09	11.37	11.38
R5	0.0299	15.52	15.17	15.34	18.26
R6	0.0225	11.65	12.23	11.84	8.60
R7	0.0225	11.70	11.63	11.79	11.67

## Data Availability

Data could be available after acceptance upon a reasonable request to the corresponding author.
